# Disparities in Overall Survival Rates for Cancers across Income Levels in the Republic of Korea

**DOI:** 10.3390/cancers16162923

**Published:** 2024-08-22

**Authors:** Su-Min Jeong, Kyu-Won Jung, Juwon Park, Hyeon Ji Lee, Dong Wook Shin, Mina Suh

**Affiliations:** 1 Department of Medicine, Seoul National University College of Medicine, Seoul 03080, Republic of Korea; smjeong.fm@snu.ac.kr; 2 Department of Family Medicine, Seoul National University Hospital, Seoul 03080, Republic of Korea; 3 National Cancer Control Institute, National Cancer Center, Goyang 10408, Republic of Korea; ara@ncc.re.kr (K.-W.J.); jwpark7@ncc.re.kr (J.P.); leehj612@ncc.re.kr (H.J.L.); 4 Department of Clinical Research Design and Evaluation, Samsung Advanced Institute for Health Science and Technology, Sungkyunkwan University School of Medicine, Seoul 06351, Republic of Korea; 5 Department of Family Medicine, Samsung Medical Center, Seoul 06351, Republic of Korea; 6 Supportive Care Center, Samsung Medical Center, Seoul 06351, Republic of Korea

**Keywords:** income disparity, cancer survival, slope index of inequality

## Abstract

**Simple Summary:**

The study analyzed income disparities in 5-year survival rates (5YSR) among cancer patients in South Korea from 2002 to 2018. Overall, 5YSR improved from 2002–2006 to 2014–2018, particularly for lung, liver, and stomach cancers. The Slope Index of Inequality (SII) indicated significant income-related disparities, with higher survival rates associated with higher incomes, notably for lung and liver cancers. The SII for lung, liver, and stomach cancer increased, while that of thyroid, breast, cervical, prostate, and colorectal cancer decreased over the study period. The findings highlight persistent income-related gaps in cancer survival despite overall improvements, emphasizing the need for targeted interventions to address socioeconomic inequalities in cancer care outcomes.

**Abstract:**

Background: The overall survival rates among cancer patients have been improving. However, the increase in survival is not uniform across socioeconomic status. Thus, we investigated income disparities in the 5-year survival rate (5YSR) in cancer patients and the temporal trends. Methods: This study used a national cancer cohort from 2002 to 2018 that was established by linking the Korea Central Cancer Registry and the National Health Insurance Service (NHIS) claim database to calculate the cancer survival rate by income level in the Republic of Korea. Survival data were available from 2002 onward, and the analysis was based on the actuarial method. We compared the survival of the earliest available 5-year period of 2002–2006 and the latest available 5-year period of 2014–2018, observing until 31 December 2021. Income level was classified into six categories: Medical Aid beneficiaries and five NHIS subtypes according to insurance premium. The slope index of inequality (SII) and relative index of inequality were used to measure absolute and relative differences in 5YSR by income, respectively. Results: The 5YSR between the 2002–2006 and 2014–2018 periods for all cancers improved. A significant improvement in 5-year survival rates (5YSR) over the study period was observed in lung, liver, and stomach cancer. The SII of survival rates for lung (17.5, 95% confidence interval (CI) 7.0–28.1), liver (15.1, 95% CI 10.9–19.2), stomach (13.9, 95% CI 3.2–24.7), colorectal (11.4, 95% CI 0.9–22.0), and prostate (10.7, 95% CI 2.5–18.8) cancer was significantly higher, implying higher survival rates as income levels increased. The SII for lung, liver, and stomach cancer increased, while that of thyroid, breast, cervical, prostate, and colorectal cancer decreased over the study period. Conclusions: Although substantial improvement in the 5YSR was observed across cancer types and income levels from 2002 to 2018, this increase was not uniformly distributed across income levels. Our study revealed persistent income disparities in the survival of cancer patients, particularly for lung and liver cancer.

## 1. Introduction

Cancer is a leading global cause of death, accounting for 10 million deaths worldwide in 2020 [1]. Because of advances in cancer prevention, diagnosis, and treatment, the global cancer mortality rate declined from 2016 to 2020 (approximately 2% annually) [2]. In Korea, cancer mortality has been decreasing since 2002, with an annual decrease of 2.7% from 2002 to 2013 and of 3.3% from 2013 to 2019 [3].

While the 5-year survival rates are improving from 42.9% in 1993–1995 to 71.5% in 2016–2020 in Korea [4], such an improvement in the cancer survival rate is not uniform across socioeconomic status. A study in Korea also demonstrated inequalities in cancer survival rates by income based on data from 2014 and 2018 [5]. Compared to the highest income group, those in medical aid had a higher death risk for stomach, colorectal, liver, and lung cancer. A large prospective US study also found a gap in all-cause survival rates by income of 80% in the most deprived group and 88% in the least deprived group [6]. A study in the US demonstrated that low- and middle-income counties had higher cancer mortality rates compared with high-income counties, and the differences were mediated by factors such as inadequate access to health care, low-quality care, and health risk behaviors [7].

Disparities in cancer survival have been increasing; for example, 11% units lower 5-year relative survival in 1987–1991 and 22% units lower 5-year relative survival in 2005–2009 were noted in the low-income group compared to the high-income group in Denmark [8]. Studies addressing updated findings and trends in disparities in cancer survival are needed. In a large cohort study in England, the survival rates for nearly all types of cancer steadily improved from 1996 to 2013 [9]. However, the difference in survival rates based on socioeconomic deprivation has remained unchanged or even increased.

The degree of disparities in cancer survival according to income levels may be different by type of cancer. A higher mortality rate ratio in the lowest income group compared with the highest income group was prominent for liver cancer (2.32), followed by stomach cancer (2.29) in men and breast cancer (2.13) in women [10]. For cancers with a poor prognosis, such as pancreatic and lung cancer, greater socioeconomic inequalities were noted [11]. However, some studies reported greater differences in the survival of cancers with a good prognosis, since the stage at diagnosis can be a more important prognostic factor for survival [12].

Therefore, in this study, we investigated the disparities in overall survival and the temporal trends among cancer patients by income group. We also examined disparities in overall survival among the eight most common cancer types in Korea: stomach, colorectal, liver, lung, thyroid, breast, cervical, and prostate cancer [4].

## 2. Materials and Methods

### 2.1. Data Source and Study Participants

This study used a national cancer cohort from 2002–2018 that was established by linking the Korea Central Cancer Registry (KCCR) and National Health Insurance Service (NHIS) claim databases to calculate the overall survival rate by income level. The survival rate of cancer patients diagnosed between 2002 and 2018 was calculated based on the results of follow-up until 31 December 2021. The KCCR data include sex; age at diagnosis; Surveillance, Epidemiology, and End Results (SEER) score; and date of cancer diagnosis. The NHIS data include death records and socioeconomic status variables such as income.

Study participants were patients diagnosed from 2002 to 2018 with the International Classification of Diseases, 10th revision (ICD-10) code of ‘C16′ (stomach cancer), ‘C18–20′ (colorectal cancer), ‘C22’ (liver cancer), ‘C33–34’ (lung cancer), ‘C50’ (breast cancer), ‘C53’ (cervical cancer), ‘C61’ (prostate cancer), or ‘C73’ (thyroid cancer). These eight cancer types were selected based on the incidence rates in Korea [3].

### 2.2. Independent Variables

In this study, the independent variable was income level, as it determines the insurance premium in the mandatory social health insurance system in Korea. The insurance premium is calculated individually (per household) according to the employee’s monthly wage or the household’s wealth (income, property, and cars). Income level was classified into six categories: Medical Aid beneficiaries (those in the lowest income bracket, exempt from insurance subscriptions, and subsidized by the government) and income quintiles of National Health Insurance (NHI) subscribers (from the 1st quintile, the lowest income, to the 5th quintile, the highest income). This definition of income levels was widely used in several studies on income and various health outcomes [13].

### 2.3. Dependent Variables

The dependent variable was a 5-year observed survival rate. Survival data for observed survival were available from 2002 onward, and the analysis was based on the actuarial (or life table) method. We compared survival in the earliest available 5-year period, 2002–2006, and the latest available 5-year period, 2014–2018. During the period from 2002 to 2018, 5-year survival rates in Korea consistently improved, as indicated by previous literature [4]. In addition, the complete approach was used to estimate 5-year survival for patients who were diagnosed with cancer more recently (2014–2018), as this approach allows prediction of survival in cases in which 5 years of follow-up are not yet available. Survival rates were age-standardized according to the International Cancer Survival Standard(ICSS) proposed by Corazziari et al. [14]. The age-standardized rate was calculated by the weighted average of the age-specific rates, where the weights represent the proportions of people in ICSS. Since the primary focus of our research was to investigate the actual survival experiences of cancer patients across different income levels in Korea, we used observed survival rather than relative survival. Observed survival provides a comprehensive measure that encompasses all causes of death, including cancer-specific mortality.

### 2.4. Analytical Approach and Statistics

In this study, frequency analysis for the number of cancer cases by type was performed. Differences in 5-year survival rates (5YSR) between the 2002–2006 and 2014–2018 periods were presented as percent point (%p) survival rates in 2014–2018 minus survival rates in 2002–2006. The slope index of inequality (SII) and relative index of inequality (RII) were estimated to assess disparities in 5YSR by cancer type [15]. The SII is an index of the predicted absolute difference in 5YSR between the Medical Aid beneficiaries and the highest income group, 5Q. The zero value of SII is interpreted as no difference in 5YSR between the lowest income group and the highest income group. A greater value of SII indicates a greater level of inequality. A positive SII value implies lower survival rates in the lower-income group. The RII was calculated as SII divided by the average 5YSR and measured as the proportion relative to the average population level. All analyses were performed using SAS statistical software (version 9.4; SAS Institute, Cary, NC, USA).

## 3. Results

### 3.1. Trend in Cancer Diagnoses: Comparison of 2002–2006 and 2014–2018

**Figure 1:**
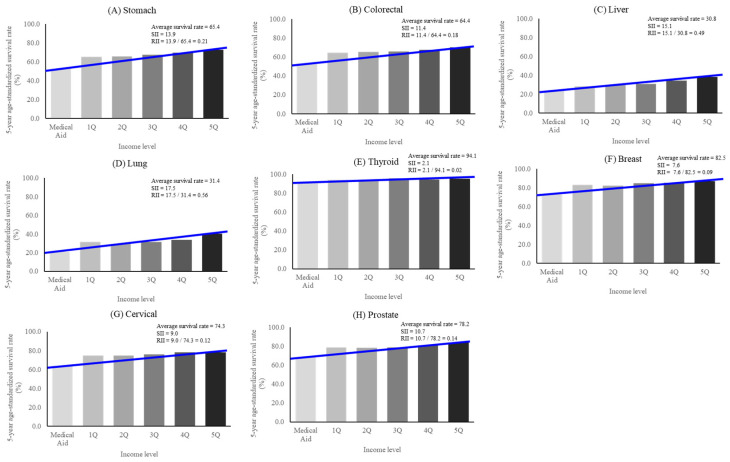
The 5-year age-standardized overall survival rates by cancer type and income level in 2014–2018. SII: Slope Index of Inequality; RII: Relative Index of Inequality.

The numbers of patients diagnosed with cancer in 2002–2006 and 2014–2018 are presented in Table 1. The number of patients with prostate cancer increased over these periods by more than three-fold, followed by the numbers of thyroid (2.61 times), breast (2.21 times), and colorectal cancer patients (1.66 times). The number of patients with cervical cancer decreased from 2002–2006 to 2014–2018 (0.85 times). The 5-year age-standardized overall survival rates by cancer type and income level in 2014–2018 are depicted in Figure 1.

### 3.2. Stomach Cancer

The overall 5YSR for stomach cancer increased from 48.0% in 2002–2006 to 68.0% in 2014–2018 in Table 2. The difference in 5YSR was greater in higher-income groups, with a 19.7%p increase in the highest income group (5Q) vs. a 14.5%p increase in the Medical Aid group. As a result, the SII of 5YSR for stomach cancer increased from 13.2 (95% CI 7.4–19.0) in 2002–2006 to 13.9 (95% CI 3.2–24.7) in 2014–2018.

### 3.3. Colorectal Cancer

The overall 5YSR for colorectal cancer increased from 56.5% in 2002–2006 to 66.4% in 2014–2018. The difference in 5YSR was greater in the 2Q income group, with an 11.8%p increase in the 2Q income group vs. an 8.9%p increase in the 5Q income group. As a result, the SII of 5YSR for colorectal cancer decreased from 14.7 (95% CI 7.3–22.0) in 2002–2006 to 11.4 (95% CI 0.9–22.0) in 2014–2018.

### 3.4. Liver Cancer

The overall 5YSR for liver cancer increased from 16.1% in 2002–2006 to 32.1% in 2014–2018. The difference in 5YSR was greater in the higher-income group, with a 17.1%p increase in the highest income group (5Q) vs. an 11.9%p increase in the Medical Aid group. As a result, the SII of 5YSR for liver cancer increased from 10.9 (95% CI 6.3–15.6) in 2002–2006 to 15.1 (95% CI 10.9–19.2) in 2014–2018, and the RII was 0.7 (95% CI 0.4–1.0) in 2002–2006 and 0.5 (95% CI 0.4–0.6) in 2014–2018.

### 3.5. Lung Cancer

The overall 5YSR for lung cancer increased from 15.1% in 2002–2006 to 33.6% in 2014–2018. The difference in 5YSR was greater in higher-income groups, with a 21.8%p increase in the highest income group (5Q) vs. 9.6%p for the medical aid group. As a result, the SII of 5YSR for lung cancer increased from 8.6 (95% CI 6.3–10.9) in 2002–2006 to 17.5 (95% CI 7.0–28.1) in 2014–2018.

### 3.6. Thyroid Cancer

The overall 5YSR for thyroid cancer increased from 89.6% in 2002–2006 to 94.7% in 2014–2018. The difference in 5YSR was greater in the lower-income group, with a 7.6%p increase in the 3Q income group vs. a 3.8%p increase in the 5Q income group. As a result, the SII of 5YSR for thyroid cancer decreased from 5.6 (95% CI 2.5–8.7) in 2002–2006 to 2.1 (95% CI −1.0–5.2) in 2014–2018.

### 3.7. Breast Cancer

The overall 5YSR for breast cancer increased from 74.8% in 2002–2006 to 84.2% in 2014–2018. The difference in 5YSR was greater in the lower-income group, with a 10.9%p increase in the 1Q income group vs. an 8.2%p increase in the 5Q income group. As a result, the SII of 5YSR for breast cancer decreased from 9.5 (95% CI 3.1–15.9) in 2002–2006 to 7.6 (95% CI −0.4–15.6) in 2014–2018.

### 3.8. Cervical Cancer

The overall 5YSR for cervical cancer increased from 73.7% in 2002–2006 to 75.9% in 2014–2018. The difference in 5YSR was greater in the lower-income group, with a 3.7%p increase in the 2Q income group vs. a 1.3%p increase in the 5Q income group. As a result, the SII of 5YSR for cervical cancer decreased from 10.2 (95% CI 3.2–17.3) in 2002–2006 to 9.0 (95% CI −1.3–19.2) in 2014–2018.

### 3.9. Prostate Cancer

The overall 5YSR for prostate cancer increased from 67.1% in 2002–2006 to 81.1% in 2014–2018. The difference in 5YSR was greater in the lower-income groups, with a 20.7%p increase in the 2Q income group vs. an 11.3%p increase in the 5Q group. As a result, the SII of 5YSR for prostate cancer decreased from 19.4 (95% CI 12.5–26.2) in 2002–2006 to 10.7 (95% CI 2.5–18.8) in 2014–2018.

## 4. Discussion

In this study, we observed an improvement in overall 5YSR across cancer types and income levels from 2002 to 2018. However, the increase in overall 5YSR was not evenly distributed across income levels. A great difference in overall 5YSR was observed for lung, liver, and stomach cancer. The SII for lung, liver, and stomach cancer increased over the period, while that for colorectal, prostate, cervical, breast, and thyroid cancer decreased over the period.

Our findings on disparities in cancer survival are consistent with previous studies. Population-based studies in Finland have shown that lower social class based on occupation is associated with poorer cancer survival rates [16]. Another Finnish study similarly reported that cancer survival was consistently higher for patients with the highest education compared to those with only basic education, primarily due to less favorable cancer stages in the lower education categories [17]. In addition to confirming the existence of disparities in cancer survival by socioeconomic status, our study also identified changes in survival disparities over the study period, including among the eight most common cancer types. Previous literature on the trends in cancer survival disparities is limited. The National Longitudinal Mortality Study in the US revealed that individuals with lower incomes experienced higher cancer mortality rates compared to their counterparts [18]. This excess risk was particularly notable for lung, colorectal, cervical, stomach, and liver cancers. Furthermore, socioeconomic disparities in mortality for overall cancer, as well as for specific cancers such as lung, prostate, and cervical, widened between 1979 and 2011. This widening disparity was primarily due to slower improvements in cancer survival among lower socioeconomic groups.

Our study observed a significant increase in the 5YSR for lung cancer over the study period, reflecting a trend seen globally [19]. This improvement can be attributed to advancements in cancer care, such as enhanced lung cancer screening programs, better availability of molecular diagnostics, and the introduction of targeted therapies and immune checkpoint inhibitors [20,21]. However, our results indicated the disparity in 5YSR by different income groups also widened during the study period (increased SII value from 8.6 in 2002–2006 to 17.5 in 2014–2018). Specifically, we found that lower-income individuals experienced a smaller relative improvement in 5YSR compared to their higher-income counterparts. The widening gap can be partly explained by several factors, including higher smoking rates [22], lower screening rates [23], and financial barriers to treatment [10] in lower-income groups, resulting in cancer diagnosis at late-stage [24] and poor survival of lung cancer patients in low-income groups.

Liver cancer survival has improved as a result of early detection and the availability of medical interventions including radiofrequency ablation and liver transplantation, aligning with our findings [25]. We found that the low-income group had a lower survival rate for liver cancer compared with the higher group, consistent with previous studies [26,27]. In addition, the disparities in 5YSR by income status increased during the study period. The SII for liver cancer increased from 10.9 (2002–2006) to 15.1 (2014–2018), whereas the RII for liver cancer decreased from 0.7 (2002–2006) to 0.5 (2014–2018). This indicates increased inequality between Medical Aid recipients and the 5Q, despite a significant improvement in overall survival during the study period. Individuals with a lower income had higher odds of late-stage liver cancer at diagnosis (odds ratio, 1.15, 95% CI 1.01–1.32) [28]. This might reflect the delayed access to treatment for underlying liver disease and suboptimal liver cancer surveillance. The chronic progression from liver cirrhosis to liver cancer can pose a long-term financial challenge to accessing healthcare, affecting financial accessibility to cancer treatment, especially for those with lower-income levels [29].

Stomach cancer survival greatly improved over the study period in Korea (20%p). These findings might be related to national cancer screening programs to detect stomach cancer in the early stages and advances in local treatment (e.g., endoscopic submucosal dissection) [30]. The Korean government has provided national screening programs for stomach cancer, including endoscopy or upper gastrointestinal series, every other year for those over 40 years old since 2002. Income disparities in the 5YSR for stomach cancer slightly increased over the study period (SII 13.2 in 2002–2006 and 13.9 in 2014–2018). Despite the relatively high participation rates for stomach cancer (about 62% in 2018), [31] patients in the higher-income group may undergo organized and opportunistic screening more often than those in lower-income groups, allowing early detection in the higher-income group [32].

In colorectal cancer, in addition to the overall increase in 5YSR over the study periods (9.9%p), the improvement in 5YSR was greater in the lower-income group than the higher-income group, resulting in a decrease in the SII value from 14.7 to 11.4. The improved 5YSR might be due to an increase in participation rates for colorectal cancer screening across income groups. However, the average annual percent change in participation rates for screening tests was higher in the lower-income group compared with the higher-income group (5.5% vs. 5.1%) [33], which at least partly explains our results.

The survival rates for prostate cancer showed income disparities, but the extent of these disparities seemed to decrease over the study period. This phenomenon can be partly explained by a ceiling effect, wherein a difference between income groups will be smaller if the survival rates approach close to 100% [34] In Korea, the serum prostate-specific antigen (PSA) screening test is not included in national cancer screening programs. At the beginning of PSA adoption in the early 2000s, patients with a higher income were more likely to undergo a screening test, leading to large disparities in 5YSR by income, but it became also popular in the lower-income population [35]. Similar findings were reported in Finland: the survival rates for prostate cancer remained stable after the introduction of PSA screening (1995–2004 and 2005–2014) for men with higher education levels; however, in the latter period after the introduction of PSA screening (2005–2014), the survival rates increased for men with a lower education level, indicating a narrowing difference in survival rates between the two groups [36]. Despite the narrowing gap, there is a disparity in the 5YSR by income. This might be partly because patients with prostate cancer also die from other cancers and cardiovascular diseases [37], which is generally higher in individuals in the lower-income group.

The improvement in overall survival rates of cervical cancer was the smallest over the study period (2.2%p). The survival rate for cervical cancer has been increasing, which is likely due to the introduction of a national cervical cancer screening program in 2002 and a national immunization program against human papillomavirus (HPV) in 2016. The national cervical cancer screening program for Medicaid beneficiaries started in 1999. Although disparities in survival rates decreased between 2002–2006 and 2014–2018 (SII from 10.2 to 9.0), income disparities exist due to inequality in cervical cancer screening participation [38] and HPV vaccine uptake [39].

The disparity in breast cancer survival by income tended to slightly decrease over the study period, partly due to increasing participation rates in breast cancer screening programs and good accessibility to standard treatments for breast cancer under universal health coverage with a 5% co-payment for cancer treatment [40]. Despite the slightly decreased disparity in breast cancer survival, an income disparity in the survival of breast cancer patients still exists. In previous studies, women with breast cancer in the lower-income group showed lower survival rates, partly explained by lower participation rates in screening, resulting in cancer diagnosis in advanced stages [41]. The overall participation rates for breast cancer screening tests (mammography) have increased from 37.6% in 2005 to 61.2% in 2015, with a 5.1% annual percentage change across income groups. However, the participation rates were lower in the lowest-income group compared with the highest-income group (53.8% vs. 68.3%) [38]. In addition, women with a medium or high household net worth were more likely to have better adherence to hormonal therapy than those with a low net worth, which might affect disparities in overall survival [42].

Since the national cancer screening program does not include screening for thyroid cancer, individuals in the high-income group had higher rates of opportunistic health screening [43]. In this context, household income was significantly associated with thyroid cancer stage at diagnosis, and stage III and IV patients had a lower income, resulting in worse survival outcomes [44,45]. In our study, the SII decreased from 5.6 in 2002–2006 to 2.1 in 2014–2018, suggesting improved income disparities for the 5YSR of thyroid cancer. Similar to PSA screening, screening for thyroid cancer with ultrasonography became more widespread in lower-income groups during the study period [43]. In addition, as the overall survival of thyroid cancer patients increased by more than 90%, the gap between lower and higher-income groups could be smaller, as mentioned above (the ceiling effect).

Our study has several limitations that should be considered. First, we did not examine information on treatments and treatment compliance, which could affect cancer survival by income levels. In addition, other socioeconomic status-related variables such as education and occupation were not examined because of a lack of information. Second, the primary outcome in our study was the 5-year observed cancer survival rate, which accounted for all-cause deaths rather than specifically focusing on cancer-specific deaths. Third, our study was performed using data from the Korean healthcare system and national health policy, and thus there is a limitation of generalizability to other countries.

## 5. Conclusions

In conclusion, substantial improvements in overall survival rates were observed across cancer types and income levels from 2002 to 2018. However, this increase in survival rates was not uniformly distributed across income levels. Our study revealed persistent income disparities in the 5YSR for cancer, especially increased income disparities for lung, liver, and stomach cancer. Identifying the multiple factors underlying these disparities and developing optimal strategies is crucial to reducing the income disparities in survival rates in future research.

## Figures and Tables

**Table 1:** The number of cancer patients by cancer type and income level.

	Study Periods							
	2002–2006	2014–2018	2002–2006	2014–2018	2002–2006	2014–2018	2002–2006	2014–2018
	Stomach		Colorectal		Liver		Lung	
Medical aid	6944	6634	4317	7162	4535	4989	5958	7650
1Q (lowest)	16,671	20,743	10,472	19,909	9109	11,065	10,252	16,089
2Q	15,506	18,948	9372	17,509	9309	10,349	9654	13,666
3Q	19,304	21,753	11,955	20,168	11,215	11,730	11,332	16,253
4Q	24,803	28,954	15,885	25,316	13,971	14,164	14,677	21,808
5Q (highest)	33,124	41,818	23,821	35,697	17,698	18,991	19,363	34,197
Total	116,352	138,850	75,822	125,761	65,837	71,288	71,236	109,663
Ratio (2014–2018/2002–2006)	1.19		1.66		1.08		1.54	
	Thyroid		Breast		Cervical		Prostate	
Medical aid	1088	2320	1767	3335	1414	887	729	2054
1Q (lowest)	5780	18,629	6419	17,044	3635	3284	1634	6613
2Q	5953	18,470	6234	15,359	3239	2917	1552	5807
3Q	7869	21,892	7601	16,138	3585	3128	2002	7094
4Q	10,930	29,681	9599	20,151	3800	3164	3132	10,889
5Q (highest)	18,166	39,068	13,652	28,107	4071	3416	6284	20,752
Total	49,786	130,060	45,272	100,134	19,744	16,796	15,333	53,209
Ratio (2014–2018/2002–2006)	2.61		2.21		0.85		3.47	

**Table 2:** Trends in 5-year age-standardized observed survival rates and disparities by cancer type, income level, and year (2002–2006 and 2014–2018) (unit: %).

	Stomach			Colorectal			Liver			Lung		
	2002–2006	2014–2018	Diff * (%p)	2002–2006	2014–2018	Diff * (%p)	2002–2006	2014–2018	Diff * (%p)	2002–2006	2014–2018	Diff * (%p)
Total	48	68	20	56.5	66.4	9.9	16.1	32.1	16	15.1	33.6	18.5
Medical aid	38	52.5	14.5	44.1	53.1	9	11.6	23.5	11.9	11.5	21.1	9.6
1Q (lowest)	44.7	65.1	20.4	52.8	64.5	11.7	13.1	28.5	15.4	12.6	31.6	19
2Q	45.4	65.4	20	53.4	65.3	11.8	13.7	29.8	16.2	12.9	29.6	16.7
3Q	46.8	67.3	20.5	54.8	65.9	11.1	14.7	30.7	16	14.6	31.7	17.1
4Q	48.6	69.3	20.7	57.8	67.7	9.9	16	34.3	18.2	15.5	33.7	18.2
5Q (highest)	52.8	72.5	19.7	61.1	70	8.9	21.1	38.1	17.1	18.6	40.5	21.8
SII (95% CI)	13.2	13.9		14.7	11.4		10.9	15.1		8.6	17.5	
	(7.4–19.0)	(3.2–24.7)		(7.3–22.0)	(0.9–22.0)		(6.3–15.6)	(10.9–19.2)		(6.3–10.9)	(7.0–28.1)	
RII (95% CI)	0.3	0.2		0.3	0.2		0.7	0.5		0.6	0.6	
	(0.2–0.4)	(0.1–0.4)		(0.1–0.4)	(0.01–0.3)		(0.4–1.0)	(0.4–0.6)		(0.4–0.8)	(0.2–0.9)	
	**Thyroid**			**Breast**			**Cervical**			**Prostate**		
	**2002–2006**	**2014–2018**	Diff * (%p)	**2002–2006**	**2014–2018**	Diff * (%p)	**2002–2006**	**2014–2018**	Diff * (%p)	**2002–2006**	**2014–2018**	Diff * (%p)
Total	89.6	94.7	5.1	74.8	84.2	9.4	73.7	75.9	2.2	67.1	81.1	14
Medical aid	84.8	92	7.2	65.6	73	7.4	65.6	63.7	−1.9	57.1	68.4	11.3
1Q (lowest)	88.6	93.9	5.3	72.3	83.2	10.9	71.4	74.5	3.2	60.7	78.7	17.9
2Q	87.5	93.4	5.8	74.1	82.1	8.1	71.2	74.9	3.7	57.7	78.4	20.7
3Q	88.1	95.6	7.6	74.6	84.7	10.1	75.9	76.1	0.1	64.7	78.9	14.2
4Q	89.9	94.6	4.7	74.8	85.1	10.3	75.5	78.3	2.8	66.9	81.1	14.2
5Q (highest)	91.5	95.3	3.8	78.8	86.9	8.2	77	78.3	1.3	72.6	83.9	11.3
SII (95% CI)	5.6	2.1		9.5	7.6		10.2	9		19.4	10.7	
	(2.5–8.7)	(−1.0–5.2)		(3.1–15.9)	(−0.4–15.6)		(3.2–17.3)	(−1.3–19.2)		(12.5–26.2)	(2.5–18.8)	
RII (95% CI)	0.06	0.02		0.1	0.09		0.1	0.12		0.3	0.1	
	(0.03–0.10)	(−0.01–0.05)		(0.04–0.2)	(−0.00- 0.19)		(0.04–0.2)	(−0.02–0.3)		(0.2–0.4)	(0.03–0.2)	

* Diff Difference between survival rate between 2002–2006 and 2014–2018; SII Slope Index of Inequality; RII Relative Index of Inequality.

## Data Availability

Data supporting the findings of this study are available from the Korean NHIS and were used under license for this study (http://nhiss.nhis.or.kr). Restrictions apply to their availability (the data are not publicly available). Data are available upon request with permission from the Korean NHIS.
